# Invasive meningococcal disease in Shanghai, China from 1950 to 2016: implications for serogroup B vaccine implementation

**DOI:** 10.1038/s41598-018-30048-x

**Published:** 2018-08-17

**Authors:** Mingliang Chen, Charlene M. C. Rodrigues, Odile B. Harrison, Chi Zhang, Tian Tan, Jian Chen, Xi Zhang, Min Chen, Martin C. J. Maiden

**Affiliations:** 1 Department of Microbiology and Division of Infectious Diseases, Shanghai Municipal Center for Disease Control and Prevention, Shanghai, China; 2 Department of Zoology, University of Oxford, Oxford, United Kingdom

## Abstract

Serogroup B invasive meningococcal disease (IMD) is increasing in China, but little is known about the causative meningococci. Here, IMD and carriage isolates in Shanghai characterised and the applicability of different vaccines assessed. Seven IMD epidemic periods have been observed in Shanghai since 1950, with 460 isolates collected including 169 from IMD and 291 from carriage. Analyses were divided according to the period of meningococcal polysaccharide vaccine (MPV) introduction: (i) pre-MPV-A, 1965–1980; (ii) post-MPV-A, 1981–2008; and (iii) post-MPV-A + C, 2009–2016. Over this period, IMD incidence decreased from 55.4/100,000 to 0.71 then to 0.02, corresponding to successive changes in meningococcal type from serogroup A ST-5 complex (MenA:cc5) to MenC:cc4821, and finally MenB:cc4821. MenB IMD became predominant (63.2%) in the post-MPV-A + C period, and 50% of cases were caused by cc4821, with the highest incidence in infants (0.45/100,000) and a case-fatality rate of 9.5%. IMD was positively correlated with population carriage rates. Using the Bexsero Antigen Sequence Type (BAST) system, fewer than 25% of MenB isolates in the post-MPV-A + C period contained exact or predicted cross reactive matches to the vaccines Bexsero, Trumenba, or an outer membrane vesicle (OMV)-based vaccine, NonaMen. A unique IMD epidemiology was seen in China, changing periodically from epidemic to hyperepidemic and low-level endemic disease. At the time of writing, MenB IMD dominated IMD in Shanghai, with isolates potentially beyond coverage with licenced OMV- and protein-based MenB vaccines.

## Introduction

The encapsulated bacterium *Neisseria meningitidis* is a leading cause of bacterial meningitis and septicaemia globally, causing over 1.2 million invasive meningococcal disease (IMD) cases annually^1^. Over 90% of IMD cases are caused by serogroups A, B, C, W, X, and Y^2^, which are defined by immunological variants of the capsular polysaccharide. Of these, five (serogroups A, C, W, X, and Y) are preventable by protein-conjugate polysaccharide vaccines^3^. Most IMD is caused by meningococci belonging to one of a limited number of hyperinvasive lineages, recognised as particular clonal complexes (ccs) by multilocus sequence typing (MLST), which are usually associated with a particular serogroup^4^.

In China, during the 1950s to 1980s, serogroup A (MenA) isolates were responsible for over 95% of cases^5^, with incidence peaking in 1967 (403/100,000)^6^. These were predominantly due to ST-5 clonal complex (cc5) and cc1^7^, and in response, a non-conjugate MenA meningococcal polysaccharide vaccine (MPV) was routinely administered from 1980 onwards^6,7^. This was followed by a decrease in MenA incidence. From 2003–2005, serogroup C hypervirulent lineage ST-4821 complex (MenC:cc4821) caused outbreaks in Anhui province^5^, leading to the predominance of MenC IMD caused by MenC:cc4821^6,8^. As a result, in 2008, a serogroup A and C bivalent MPV was introduced into the vaccination program^6^, followed by an overall IMD incidence decrease to 0.047/100,000, although this may have been underestimated^7^. From 2011 onwards, the proportion of MenC IMD began to decrease while MenB increased from 7.2% in 2006 to 26.5% in 2014 nationwide^7,9^, with a few regional MenW:cc11 cases^10^.

Prevention of MenB IMD is challenging due to the poorly immunogenic serogroup B capsular polysaccharide and concerns about autoimmunity, due to its structural similarity to structures occurring on human tissue. To address this deficit, two protein-based vaccines, Bexsero (4CMenB) and Trumenba (bivalent rLP2086), have been developed and licensed in Europe and the USA^11,12^. Bexsero is composed of factor H binding protein (fHbp), Neisserial heparin-binding antigen (NHBA), *Neisseria* adhesin A (NadA), and outer membrane vesicles (OMVs) containing the porin PorA (meningococcal subtyping antigen) as a major immunogen, whereas Trumenba contains two fHbp-subfamily variants^13^. Both Bexsero and Trumenba may elicit protective responses across meningococci of diverse serogroup expressing these or related protein variants^14^. Two methods have been established to predict Bexsero coverage. (i) The Meningococcal Antigen Typing System (MATS) combines phenotypic and functional assays^12^; however, it is time and labour intensive, requires toddler serum, and is only performed by specialist laboratories. (ii) The Bexsero Antigen Sequence Typing (BAST) which uses sequence data is a rapid, scalable, and portable genotypic approach, which catalogues deduced peptide sequences and matches to vaccine variants (BAST-1) or cross-reactive variants^15^. The meningococcal antigen surface expression (MEASURE) system is used to assess Trumenba coverage by flow cytometry^16^.

Limited information is available documenting *N. meningitidis* isolates associated with IMD and carriage in China over the past 60 years. In this study, fluctuations of IMD and meningococcal carriage are described in association with the introduction of MPV vaccines in Shanghai, China since 1950. In addition, and in response to increasing MenB IMD^7^, we assessed the potential impact of protein-based vaccines to local prevalent serogroups and clonal complexes.

## Results

### Epidemiology and characterisation of *N. meningitidis* isolates associated with IMD and carriage in Shanghai

**Figure 1:**
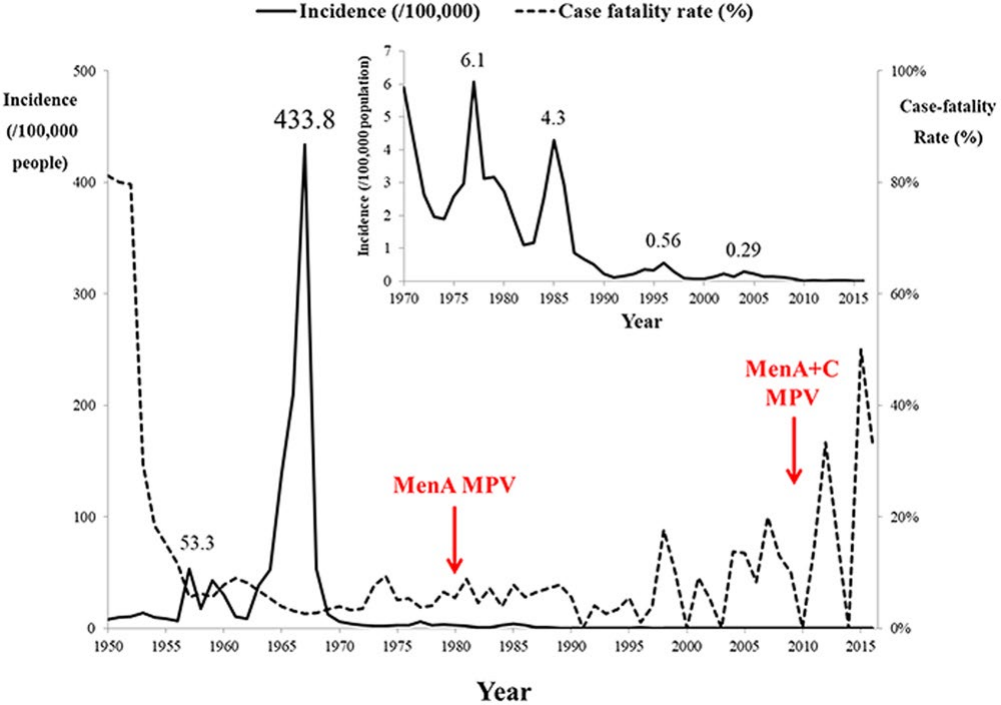
Invasive meningococcal disease incidence in Shanghai, China during 1950–2016, as reported in National Notifiable Diseases Registry System. The times of introduction of serogroup A (1980) and serogroups A and C polysaccharide vaccines (2008) in Shanghai, China were indicated with red arrow. Inset figure shows the incidence after 1970. The highest incidences in different epidemic period were labelled. MenA, serogroup A meningococcus; MPV, meningococcal polysaccharide vaccine.

**Figure 2:**
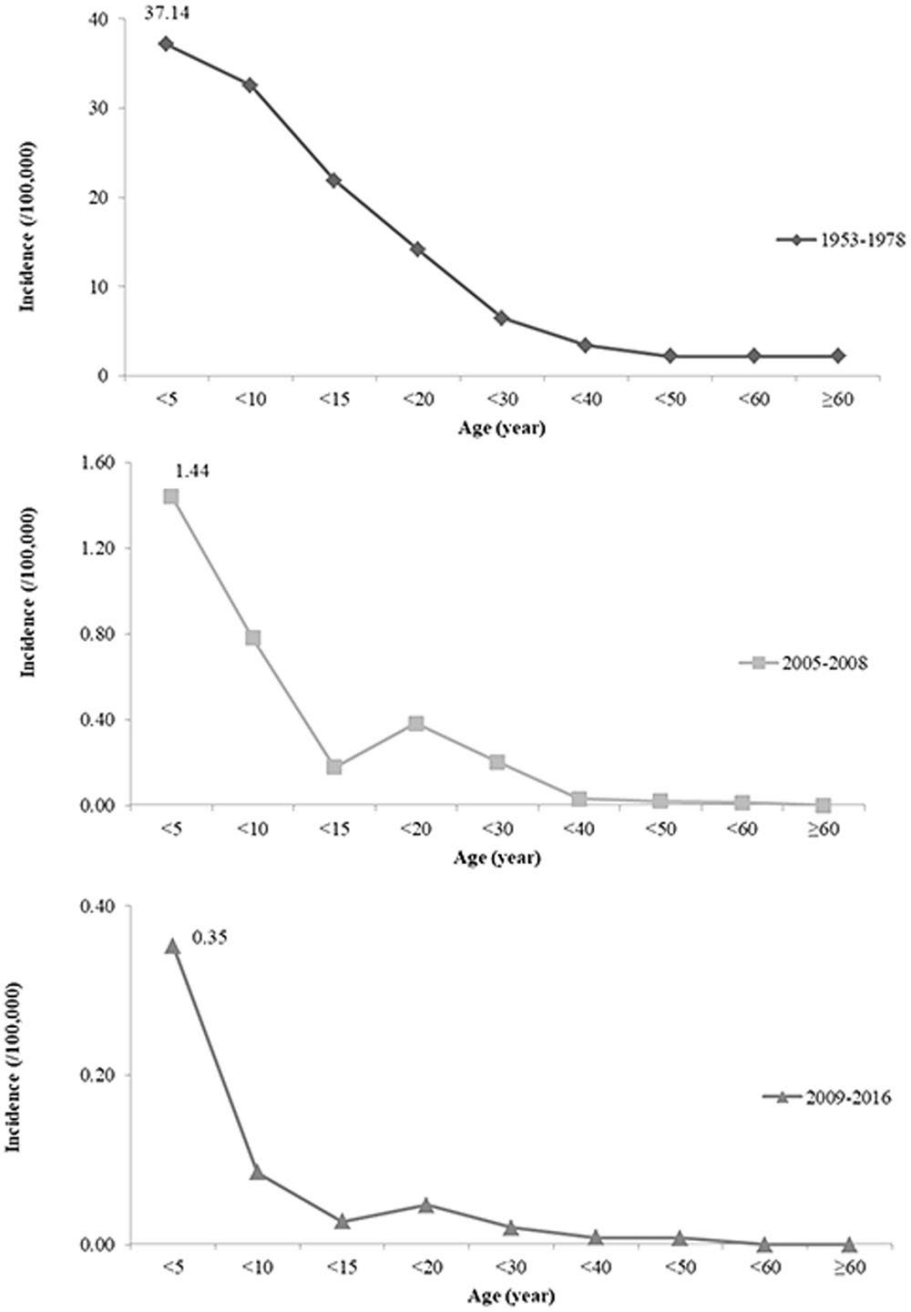
Analysis of invasive meningococcal disease incidence in Shanghai, China by age group.

**Figure 3:**
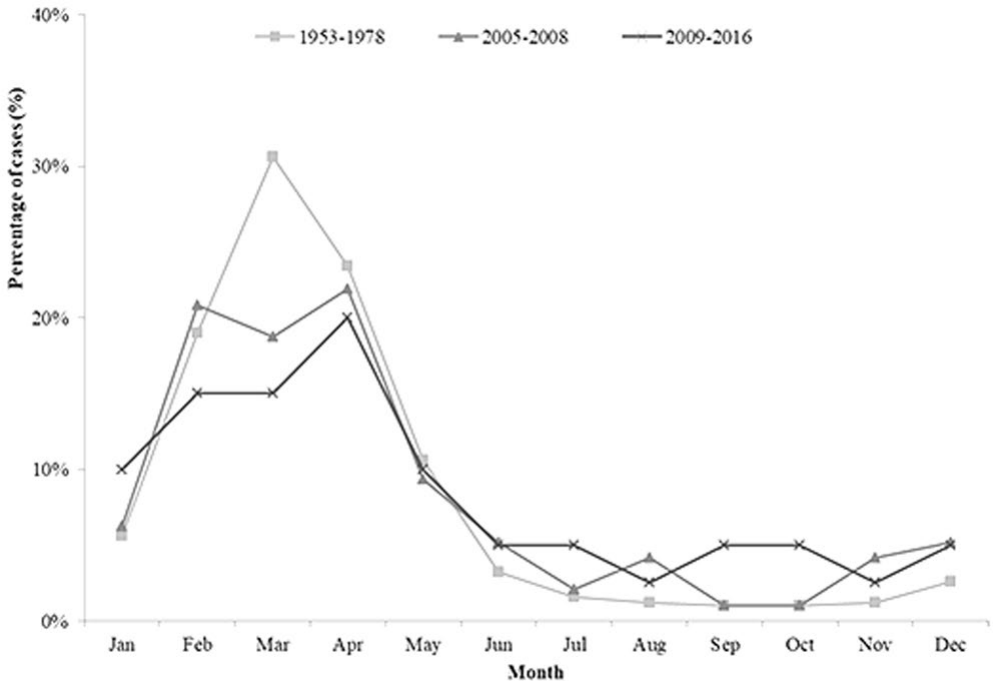
Seasonality of invasive meningococcal disease in Shanghai, China.

**Figure 4:**
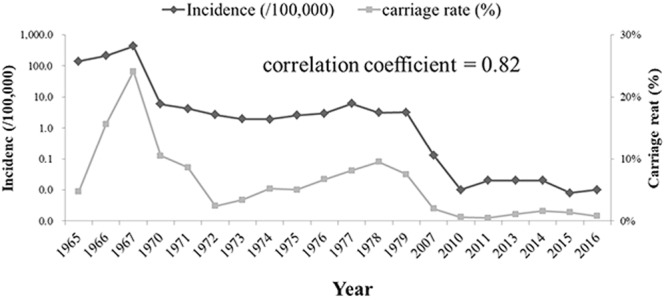
Relationship between invasive meningococcal disease incidence and meningococcal carriage rate observed in Shanghai, China.

From 1950 to 2016, seven IMD epidemic periods were observed, each lasting 8–10 years (Fig. 1). Average incidence per 100,000 population was: 1953–1961, 21 (case-fatality rate, 8.5%); 1962–1972, 87 (3.3%); 1973–1981, 2.9 (5.7%); 1982–1990, 1.6 (6.3%); 1991–2000, 0.23 (3.7%); 2001–2008, 0.17 (9.8%); and 2009–2016, 0.02 (15%). Highest incidence occurred in children aged <5 years, decreasing with age, except in those aged 15–19 years from 2005 (Fig. 2). Seasonality of IMD rates was apparent: 50–70% of cases occurred between February and April, with fewer cases (8–23%) between June and October (Fig. 3). A positive correlation was observed between observed carriage rate and IMD incidence (Fig. 4).

**Figure 5:**
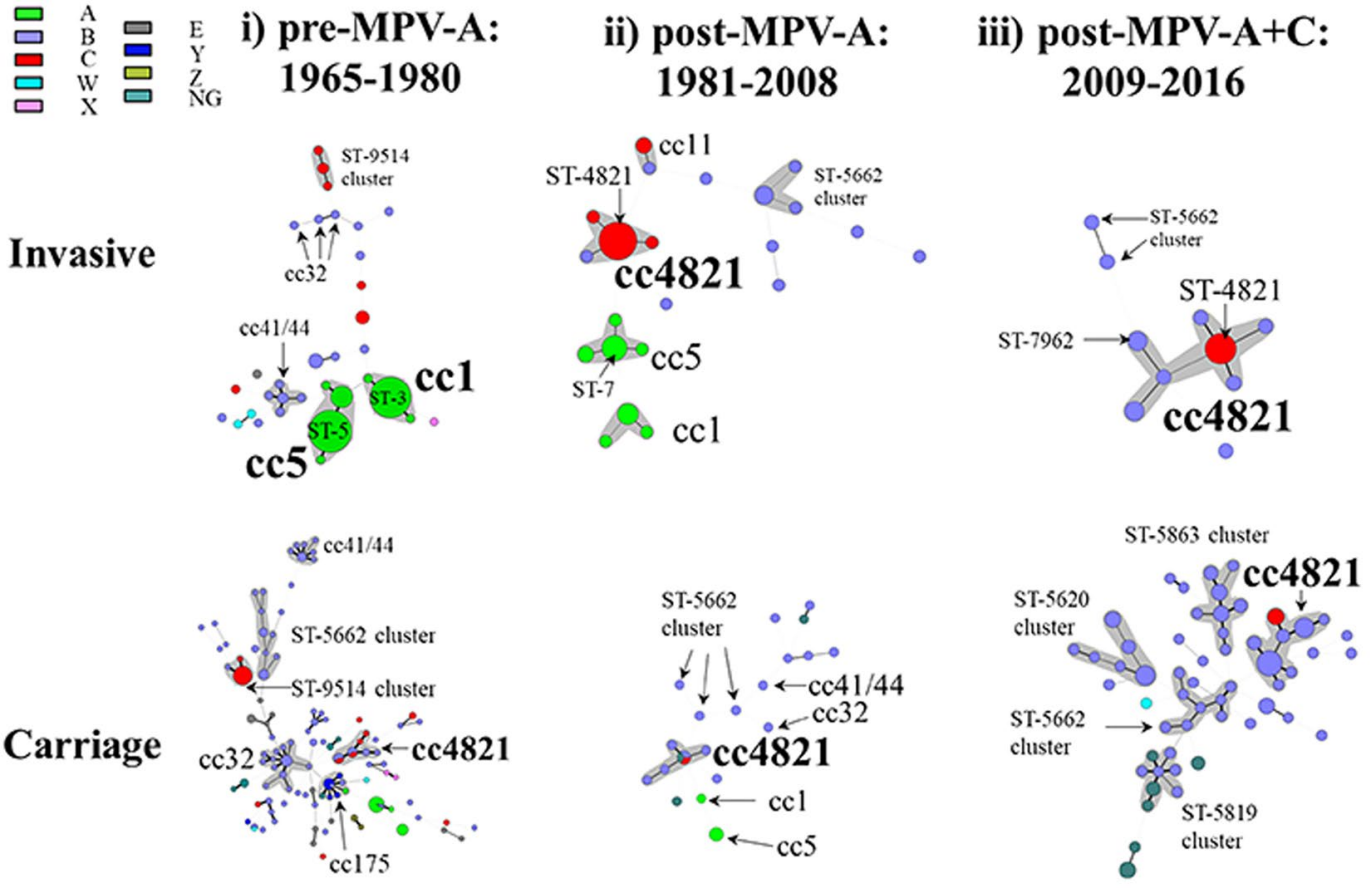
Minimum-spanning tree analysis of multilocus sequence types of invasive and carriage N. meningitidis before and after introduction of meningococcal vaccines in China. Isolates were obtained during the pre-MPV-A (1965–1980), post-MPV-A (1981–2008), and post-MPV-A + C (2009–2016) periods. Sequence types (STs) are displayed as circles. The size of each circle indicates the number of isolates with this particular type. Serogroup is distinguished by different colours. The shaded halo surrounding the STs encompasses related sequence types that belong to the same clonal complex. Heavy solid lines represent single-locus variants, and light solid lines represent double-locus variants. Sequences types sharing no less than 4 loci, but not assigned to any clonal complexes in the PubMLST database were assigned to ST-clusters. NG, nongroupable.

Based on the time of introduction of MPVs in China (1980 and 2008), three periods were defined: (i) pre-MPV-A, 1965–1980; (ii) post-MPV-A, 1981–2008; and (iii) post-MPV-A + C, 2009–2016 (Table 1 and Fig. 5).

(i) In the pre-MPV-A period, the average IMD incidence was 55.4/100,000. MenA isolates were predominant (84/117, 71.8%; Table 1), with most belonging to cc5 (48/84, 57.1%) and cc1 (36/84, 42.9%). Among MenA:cc5 isolates, ST-5 prevailed (37/48, 77.1%), with no ST-7 observed. All ST-5 contained PorA P1.20,9, while of the MenA:cc1 isolates, 34/36 (94.4%) were ST-3, with 32/34 (94.1%) P1.7-1,10. MenB isolates were assigned to: cc41/44 (6/20, 30%); cc32 (3/20, 15%); cc8 (1/20, 5%); cc35 (1/20, 5%); and cc198 (1/20, 5%), with eight STs unassigned to a cc. MenC isolates were assigned to ST-9514 cluster (44.4%, 4/9; Fig. 5 and S1), cc4821 (3/9, 33.3%), and cc231 (1/9, 11.1%). MenC:cc4821 isolates were all ST-3436 with P1.20-3,23 several variants including P1.20-3,23-1 and P1.20-3,23-3. The observed meningococcal carriage rate ranged from 24.1% in 1967 to 2.4% in 1972 (Table S1), with overall carriage rates of 4.4% (368/8,319) in children and 9.9% (888/8,956) in adults (≥15 years). In 1966–1967, high IMD incidence (>200/100,000) coincided with high carriage rates (>15%), of which a high proportion (>70%) was MenA. This decreased from 50% in 1970 to 1.1% in 1979. Among the 178 carriage isolates analysed, MenB (52.2%) was predominant (Table 1), with cc32 (17/93, 18.3%) the most prevalent.

**Table 1:** Epidemiological information and molecular characterisation of meningococcal isolates before and after introduction of vaccines in Shanghai, China

Period	Disease isolates			Carriage isolates		
	i) pre-MPV-A^§^:	ii) post-MPV-A:	iii) post-MPV-A + C:	i) pre-MPV-A:	ii) post-MPV-A:	iii) post-MPV-A + C:
	1965–1980	1981–2008	2009–2016	1965–1980	1981–2008	2009–2016
	(n = 117)	(n = 61)*	(n = 19)^†^	(n = 178)	(n = 24)	(n = 89)
Incidence, /100,000	55.4 (range, 1.9–433.8)	0.71 (0.06–4.3)	0.02 (0.008–0.03)	9.3% (carriage rate, 2,832/30,766)	2.0% (11/553)	1.2% (83/6,284)
Case fatality rate%	3.0 (2,918/97,280)	6.5 (168/2,580)	15 (6/40)	NA^¶^	NA	NA
Serogroup	A (71.8%, 84), B (17.1%, 20), C (7.7%, 9)	A (29.5%, 18), B (24.6%, 15), C (45.9%, 28)	A (0%), B (63.2%, 12), C (36.8%, 7),	A (10.7%, 19), B (52.2%, 93), C (16.9%, 30),	A (16.7%, 4), B (66.7%, 16), C (4.2%, 1)	A (0%) B (84.3%, 75), C (3.4%, 3),
Clonal complex^♀^	cc5 (41.0%, 48), cc1 (30.8%, 36)	cc4821 (37.5%, 18/48)^‡^, cc5 (20.8%, 10/48)	cc4821 (75%, 12/16)	cc32 (9.6%, 17), cc4821 (8.4%, 15), cc5 (7.3%, 13)	cc4821 (29.2%, 7)	cc4821 (25.8%, 23)
PorA VR	P1.20,9 (41.0%, 48), P1.7-1,10 (29.1%, 34)	P1.7-2,14 (29.2%, 14/48), P1.20,9 (20.8%, 10/48)	P1.7-2,14 (43.8%, 7/16)	P1.7-4,13-20 (11.2%, 20), P1.7,16 (9.0%, 16), P1.20,9 (7.9%, 14)	P1.7-2,14 (12.5%, 3), P1.20,9 (12.5%, 3)	P1.21-2,28 (13.5%, 12), P1.22,23-3 P1.18-25,9-18 (5.6%, 5), P1.22,23 (5.6%, 5) (6.7%, 6)
FetA VR	F3-1 (32.5%, 38), F5-5 (31.6%, 37),	F3-3 (34.2%, 13/38), F3-1 (23.7%, 9/38)	F3-3 (42.9%, 6/14)	F5-8 (11.8%, 21), F1-7 (10.1%, 18), F1-15 (9.0%, 16), F3-1 (6.7%, 12)	F1-5 (12.5%, 3), F3-1 (12.5%, 3), F3-3 (12.5%, 3)	F1-20 (13.5%, 12), F1-91 (10.1%, 9)
BAST	13 (38.5%, 45), 794 (29.1%, 34)	22 (21.1%, 8/38), 802 (15.8%, 6/38)	802 (21.4%, 3/14)	2300 (9.6%, 17/162) 13 (6.2%, 11/178)	22 (12.5%, 3/24)	2262 (5.6%, 5), 2433 (4.5%, 4)

^§^MPV-A, serogroup A meningococcal polysaccharide vaccine.

*13 isolates or positive DNA not available for multi-locus sequence typing and PorA VR, and another 10 isolates not available for typing of FetA and BAST.

^†^3 isolates or positive DNA not available for multi-locus sequence typing and PorA VR, and another 2 isolates not available for typing of FetA and BAST.

^¶^NA, not applicable.

^‡^The denominator is indicated when it is different from the total number of isolates in this period.

^♀^cc1, ST-1 complex; cc5, ST-5 complex; cc32, ST-32 complex; cc4821, ST-4821 complex.

(ii) In the post-MPV-A period, the average IMD incidence was 0.71/100,000. Based on 61 IMD cases with available serogroup data, MenC 28/61 (45.9%) was the most frequent, in which isolates belonging to cc4821 (17/19, 89.5%) dominated, with the majority of these ST-4821 (15/17, 88.2%) and P1.7-2,14. MenA:cc5 (10/16, 62.5%) dominated the MenA isolates: of the eight collected during 2005–2008 6/8 (75%) were ST-7 and P1.20,9, and two isolated in 1985 were ST-5, P1.20,9. MenB isolates were assigned to cc4821 1/7 (14.3%) (ST-5798:P1.10,13-1), cc41/44 (1/7, 14.3%), and cc32 (1/7, 14.3%), with four isolates unassigned to a cc. The carriage rate from the 2007 survey was 11/553 (2.0%), with 9/369 (2.4%) in children and 2/184 (1.1%) in adults (15–46 years). MenB (16/24, 66.7%) was predominant in carriage (Tables 1), 31.3% of which belonged to cc4821, with 5 different STs each possessing a different PorA VR type.

(iii) In the post-MPV-A + C period, the average IMD incidence was 0.02/100,000. MenB (63.2%) isolates predominated, 50% of which were cc4821 and assigned to 5 STs each with a different PorA VR type (Fig. 5). All 7 MenC isolates were assigned to cc4821. Except one DNA sample with incomplete ST, other 6 MenC:cc4821 isolates were ST-4821, with 5 exhibiting PorA P1.7-2,14. The carriage rate ranged from 0.5% in 2011 to 1.6% in 2014, with 25/1,660 (1.5%) in children and 73/4,624 (1.6%) in adults (15–78 years). MenB (75/89, 84.3%) was the most frequent serogroup in carriage (Table 1), with 20/75 (26.7%) cc4821.

### Features and seasonality of MenB IMD

From 1965 to 2016, 34/47 (72.3%) of all MenB IMD occurred in children, while 60/150 (40%) of non-B IMD cases occurred in this age group (p = 0.01). Since 2005, all MenB IMD cases were in children (19 days to 12 years), among which 13/20 (65%) were infants. During 2005–2008, MenB IMD incidence was 0.01/100,000, highest among infants (1.1/100,000) compared to 0.009/100,000 in children aged 1–15 years, with no reported deaths. During the post-MPV-A + C period, MenB IMD incidence was 0.007/100,000, the highest of which in infants (0.45/100,000) compared to 0.03/100,000 in children aged 1–15 years, with a case-fatality rate of 2/21 (9.5%). During the post-MPV-A + C period, MenB cases were observed from February to September, and in December while all MenB cases from 2005–2008 occurred from January to June.

### BAST identification, prevalence of vaccine antigens and potentially cross-reactive variants

A total of 243 BASTs were identified with high diversity in each of the vaccine antigens: fHbp, 64 variants; NHBA, 95; NadA, 9 (the *nadA* gene was absent or had gene-silencing frameshift mutations in 367/460 or 82.0% of isolates); PorA VR1, 38; and PorA VR2, 64. A total of 56 BASTs were identified in the 169 IMD isolates (0.33 BASTs/isolates). The four most prevalent BASTs were BAST-13 (associated with cc5), BAST-794 (cc1), BAST-802 (cc4821) and BAST-22 (cc5), representing by 102/169 (60.4%) of the meningococcal disease isolates. In the 291 carriage isolates, 201 BASTs were identified (0.69 BASTs/isolates). The four most prevalent BASTs, BAST-2300 (ST-9514 cluster), BAST-13 (cc5), BAST-794 (cc1), and BAST-2262 (ST-5620 cluster), were represented by 40/267 (15.0%) of the isolates. BASTs fluctuated with ccs found in the pre- and post-MPV periods (Table 1).

**Figure 6:**
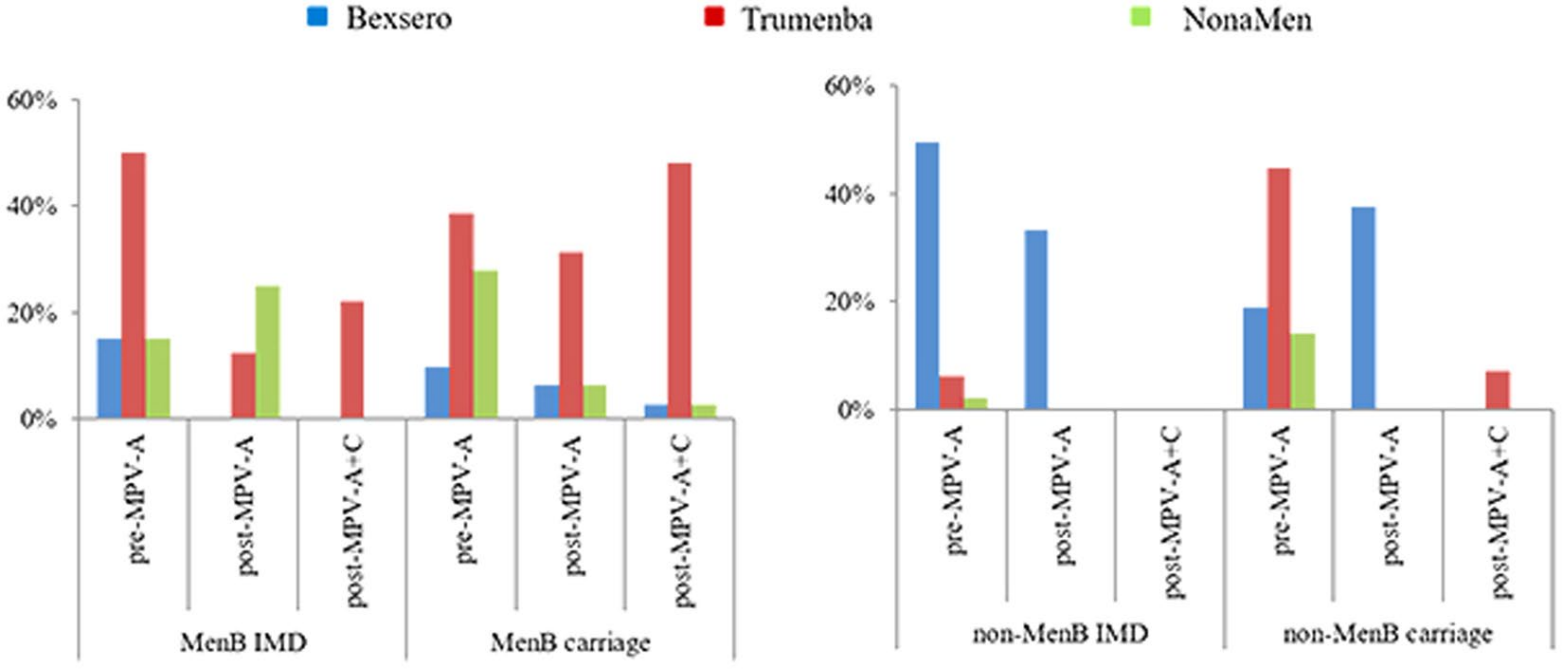
Prevalence of peptide variants, and potentially immunologically cross-reactive variants, for three serogroup B-substitute vaccines (Bexsero, Trumenba, and NonaMen) among 460 invasive and carriage meningococci from Shanghai, China in the pre-MPV-A, post-MPV-A, and post-MPV-A + C periods. Bexsero and Trumenba are two protein-based serogroup B substitute meningococcal vaccines, which have been licensed in Europe and the USA, while NonaMen is a 9-valent investigational outer membrane vesicle (OMV) vaccine, which has undergone pre-clinical testing. Three periods were defined, pre-MPV-A (1965–1980), post-MPV-A (1981–2008), and post-MPV-A + C (2009–2016), according to the time of two meningococcal polysaccharide vaccines introduced in China (1980 serogroup A, 2008 A and C).

Combined exact matches and putative cross-reactive antigens, revealed that 15/221 (6.8%) of MenB isolates were potentially covered by Bexsero, and among IMD MenB isolates, these constituted: 3/20 (15%) in pre-MPV-A; none in post-MPV-A; and none in post-MPV-A + C periods (Fig. 6). For Trumenba, no exact antigen match was found, and putative cross-reactive variants were 90/221 (40.7%) among MenB isolates. In IMD MenB isolates this constituted: 10/20 (50%); 1/8 (12.5%), and 2/9 (22.2%) in each respective period. For NonaMen, the prevalence of vaccine antigens among MenB isolates was 34/221 (15.4%), and in IMD MenB isolates, 3/20 (15%), 2/8 (25%), and none, respectively.

The prevalence of homologous and potentially cross-reactive peptide variants was noted for the 5 most prevalent ccs, which corresponded to 238/460 (51.7%) of the IMD meningococcal isolates, namely: cc1; cc5; cc32; cc41/44; and cc4821. With the exception of cc5 (73/74, 98.6%) the prevalence of such variants for Bexsero was low: cc1, none; cc4821, none; cc32, 1/23 (4.3%); and cc41/44, 9/21 (42.9%). Prevalence of similar antigens for Trumenba was: cc32, 5/23 (21.7%); cc4821, 29/72 (40%); cc41/44, 15/21 (71.4%); cc1 and cc5 < 2%. No NonaMen antigens were observed in isolates belonging to cc1, cc5, cc41/44 or cc4821, while 16/23 (69.6%) of cc32 isolates contained homologous PorA sequences. For cc4821 isolates, Trumenba contained homologous or potentially cross-reactive antigen sequences among IMD cc4821 isolates as follows: none in pre-MPV-A; 1/14 (7.1%) in post-MPV-A; and 2/10 (20%) in post-MPV-A + C periods. Among carriage cc4821 isolates, this was 10/15 (66.7%), 1/7 (14.3%) and 15/23 (65.2%), respectively.

## Discussion

This study provides a comparative analysis of IMD and meningococcal carriage isolates obtained in Shanghai, China, and the likely impact of novel vaccines on disease and carriage. From 1965 to 1980, IMD in China was dominated by MenA meningococci belonging to the hyperinvasive lineages corresponding to cc5 (ST-5) and cc1 (ST-3), resulting in several epidemics (Figs 1 and 5), consistent with similar MenA epidemics seen elsewhere^17,18^. Introduction of serogroup A MPV vaccine in China in 1980 was followed by a decrease in IMD; however, this in turn may have contributed to expansion of MenC:cc4821^5^. Clonal expansion following vaccine implementation was further observed with the implementation of serogroup A and C MPV in 2008 which was followed by an increase in MenB IMD, largely due to MenB:cc4821 isolates (Figs 1 and 5, Table 2)^19^. These data indicate that vaccine intervention may have promoted the emergence of different IMD-associated meningococci, consistent with the fluctuation of hyperinvasive lineages in the carried population; indeed, similar changes subsequent to vaccine implementation with MPV A + C were observed in Egypt and Morocco during 1992–1995^20^.

**Table 2:** Comparison of molecular characterisation of ST-4821 complex by serogroup*.

Clonal complex	Sequence type	fHbp VR	NHBA VR	PorA VR	PorB VR	FetA VR
MenB:cc4821 (n = 40)	ST-5664 (9), ST-5798 (6), ST-3200 (6)	16 (23)	669 (13), 910 (8)	P1.20,23-x (26)^¶^	3-229 (12), 3-81 (8), 3-460 (5), 3-48 (4)	F1-91 (12), F3-9 (6), F5-2 (5)
MenC:cc4821 (n = 32)	ST-4821 (23)	80 (9), 22 (6), 404 (5), 419 (5)	503 (23)	P1.7-2,14 (15), P1.20,23-x (8)	3-48 (22)	F3-3 (23)

*All cc4821 isolates without *nadA* gene.

^¶^P1.20,23-x, refers to the following nine PorA variants found in this complex: P1.20-3,23, P1.20-3,23-1, P1.20-3,23-2, P1.20-3,23-3, P1.20-3,23-6, P1.20-3,23-7, P1.20-3,23-9, P1.20-3,23-19, and P1.20-3,23-28.

Since the 1950s, the seasonal peak of IMD cases in Shanghai has been from February to April (Fig. 3), similar that seen nationally in China^6^. China is a large country, with differences seen for example in the peak influenza season between northern (January) and southern China (from June to July), with the latter characterised by a warmer, more humid climate^21^. This suggests that, besides climate and influenza incidence, national social factors should be considered when deploying preventative strategies. IMD outbreaks, such as the MenA:cc5 global pandemic and MenW:cc11 transmission, are often associated with mass gathering events^17,22^. For example, the 1967 MenA epidemics across China (403/100,000) occurred following the National Great Networking event during 1966–1967^6,23^, which involved gatherings of millions of students from all over the country^23^. The observed seasonal peaks in IMD incidence (Fig. 3) may be associated with the Spring Festival travel rush for the Chinese New Year where, from January to March, over 200 million people travel across China for family reunions^24^. Poor sanitary conditions and overcrowded environments on public transport is likely to facilitate transmission of meningococci, indicating the need for enhanced understanding of meningococcal carriage before, during, and after the Chinese New Year.

Few carriage surveys have been undertaken in China; however, two studies in the Shandong and Guangxi provinces identified high carriage rates of meningococci from hyperinvasive ccs in association with IMD outbreaks^10,25^. This is consistent with results from our study where carriage rates positively correlated with incidence (Fig. 4 and Table 1). In addition, MenB cases were not linked to a distinct seasonal pattern such that in the post-MPV-A + C period, MenB cases were found to occur throughout the year in contrast to that seen in the 1950s (Fig. 3).

Since the 1980s, three monovalent OMV-based MenB vaccines have been licensed for IMD epidemics, but these demonstrated clinical efficacy only against homologous meningococci^13^. Although a nonavalent OMV-based MenB vaccine has been evaluated^26^, the vaccine variants were found low prevalence among Chinese MenB IMD meningococcal isolates in this study (<5%) and from 27 provinces of China (<11%)^19^. Two protein-based MenB substitute vaccines have been licensed and implemented in vaccination interventions in Europe and the USA^11,12^. The coverage of MenB isolates by Bexsero in the UK during 2014/15 was predicted to be 60.8% using BAST^15^, and 66% by MATS^27^. For Trumenba, coverage rates of 78–100% to collections of diverse strains in Europe and the USA was estimated using serum bactericidal assay^28–30^. In this study, the prevalence of meningococcal antigens potentially covered by the variants present in the vaccine was low in Shanghai for both Bexsero (≤15%) and Trumenba (≤50%). Based on fHbp data from 30 provinces across China^31^, Trumenba was predicted to potentially cover 32.5% of IMD and 40% of MenB carriage isolates. This low prevalence is due to the predominance of cc4821 meningococci in China^19^, which share no homologous antigens with Bexsero and few with Trumenba (40.3%). In Europe and the USA, MenB IMD has been mainly caused by cc32, cc41/44, and cc269 over the past 20 years^32^, which exhibit distinct antigenic profiles to those found in China (Table 1). Therefore, the likely impact of Bexsero on Chinese cc32 (4.3%) and cc41/44 (42.9%) was lower than in Europe (93–100%)^27^. Alternative vaccination approaches include an OMV-based vaccine specific for MenB cc4821, and the characterisation of ST and antigen data, especially PorA variants, reported here will be invaluable in assessing vaccine coverage and future serogroup B-substitute vaccine development in China.

The interpretation of these data is limited by incomplete records of MenB IMD cases during 1950–2004 and the small number of isolates collected during 1965–1980 and 1981–2008; however, data from children’s hospitals in Shanghai and Beijing provide some insight into MenB IMD during 1976–2002. In Shanghai, from 1976 to 1985, 33.3% (20/60) of paediatric IMD cases were caused by MenB, with 45% (9/20) in infants (<1 year) and a fatality rate of 10% (2/20). During 1984–1993, 42% (21/50) of IMD cases were due to MenB, with 71.4% (15/21) occurring in children aged ≤2 years^33,34^. In Beijing, during 1984–2002, 51% (102/200) of paediatric IMD cases were caused by MenB with 51.0% (52/102) in infants and a fatality rate of 11.8% (12/102)^35^. Among these MenB cases in Shanghai and Beijing, 59.2% (84/142) occurred outside the “epidemic season” (from February to April). Therefore, it is likely the two features of MenB IMD, namely (i) the occurrence in young children and (ii) lack of seasonal variation in incidence, have persisted since the 1970s.

Our data suggest that vaccine coverage of MenB meningococci in Shanghai by licensed OMV and protein-based ‘serogroup B substitute’ MenB vaccines may be limited. Therefore, a cautious, region-specific approach to implementation of new protein-based meningococcal vaccines should be considered. Further, the temporal analysis suggests that vaccine implementation coinciding with the start of the calendar year, so as to disrupt transmission events during Spring Festival might have the highest impact on IMD incidence. In conclusion, our data indicate that IMD surveillance in China should be enhanced, combined with comprehensive carriage studies to assess the impact of vaccines and their likelihood inducing both effective direct protection and herd immunity.

## Methods

### IMD surveillance

IMD surveillance in Shanghai, implemented in the National Notifiable Diseases Registry System (NNDRS), began in 1950 and was based on monthly paper reports. Since 2004, it has become a web-based, real-time system^6^. All clinical specimens and meningococcal isolates from suspected IMD cases in Shanghai are sent to Shanghai CDC when they are reported in the NNDRS^6^. In China, a child is defined as an individual <15 years of age and an infant <1 year^6^.

### *N. meningitidis* carriage surveys

Twenty carriage studies were conducted during 1965–2016. In each study, three districts were chosen, including urban, suburban, and rural districts. Posterior oropharyngeal swabs were collected from preschool children (toddlers aged 3-6 years in childcare centres), students (aged 6-14 years in schools), and adults (staff in department stores, railway stations, army, and residents in communities), and cultured as previously described^36^.

### Isolate collection

From 1965–2016, 460 meningococcal isolates were collected in Shanghai, excluding the period 1986–2004 when isolates were not stored. As a result, 169 IMD and 291 carriage isolates dating from 1965–1985 (n = 306) and 2005–2016 (n = 154) were available for study. Serogroup was determined by slide agglutination using monoclonal antiserum (BD, USA) and PCR detection of the appropriate capsule synthesis gene^37^. Isolate serogroup distribution was: A, 123 isolates; B, 221; C, 62; E, 13; W, 5; X, 3; Y, 9; and Z, 3; and 21 nongroupable (NG, negative by PCR and sera agglutination). Sequence type (ST), cc, *porA* and *fetA* variants were determined from nucleotide sequences by querying the PubMLST.org/Neisseria database^38^. Relationships between STs were analysed using BioNumerics software package (version 7.6.2; Applied Maths, Belgium).

### BAST identification and vaccine coverage estimates

BAST was determined as described previously^15^. Briefly, genome sequences were used to deduce peptide sequences by *in silico* translation of the nucleotide sequences of *fhbp, nhba, nadA*, and *porA* variable regions 1 and 2 (VR1 and VR2). Variant numbers were assigned to unique peptide sequences using the established nomenclatures^39–41^. Each unique combination of the 5 antigenic variants was assigned a BAST number, in order of discovery; BAST-1 refers to the Bexsero vaccine constituents: fHbp 1, NHBA 2, NadA 8, and PorA P1.7–2,4. Exact matches and potential cross-reactive matches were combined to evaluate coverage of Bexsero, Trumenba, and NonaMen, a 9-valent OMV-based vaccine (Table S2)^26,28–30,42,43^.

### Statistical analysis

Statistical analysis was performed using SPSS (version 20.0; IBM, USA). Fisher’s exact test was used to compare proportions of IMD occurring in children with causative serogroup. Statistical significance was assessed at *p* < 0.05. The correlation coefficient between carriage rate and IMD incidence was calculated using Microsoft Excel 2010.

### Ethical Aspects

All the specimens from meningococcal patients and carriers were collected as part of the routine clinical management of invasive meningococcal cases, according to the national guidelines in China. Informed consent has been obtained, including that from a parent and/or legal guardian of the participants under the age of 18 years. All these experimental protocols were approved by the Institutional Review Board of Huashan Hospital, Fudan University.

### Data Availability

All data generated or analysed during this study are included in this published article and its Supplementary Information files.

## Electronic supplementary material

Supplementary material
