# Author Correction: Invasive meningococcal disease in Shanghai, China from 1950 to 2016: implications for serogroup B vaccine implementation

**DOI:** 10.1038/s41598-018-31649-2

**Published:** 2018-09-04

**Authors:** Mingliang Chen, Charlene M. C. Rodrigues, Odile B. Harrison, Chi Zhang, Tian Tan, Jian Chen, Xi Zhang, Min Chen, Martin C. J. Maiden

**Affiliations:** 1 Department of Microbiology and Division of Infectious Diseases, Shanghai Municipal Center for Disease Control and Prevention, Shanghai, China; 2 Department of Zoology, University of Oxford, Oxford, United Kingdom

Correction to: *Scientific Reports* 10.1038/s41598-018-30048-x, published online 17 August 2018

This Article contains typographical errors in the Acknowledgements section.

“the 4th Three-year Action Plan for Public Health of Shanghai (15GWZK0101) from Shanghai Municipal Commission of Health and Family Planning”

should read:

“the 4th Three-year Action Plan for Public Health of Shanghai (GWTD2015S01) from Shanghai Municipal Commission of Health and Family Planning”

