# Supplementary material for: Invasive meningococcal disease in Shanghai, China from 1950 to 2016: implications for serogroup B vaccine implementation
Source: Sci Rep. 2018 Aug 17;8:12334. doi: 10.1038/s41598-018-30048-x (PMC6098053; doi:10.1038/s41598-018-30048-x)
Supplement: Supplementary file 1 — Supplementary material [file 41598_2018_30048_MOESM1_ESM.pdf]

Supplementary material

Invasive meningococcal disease in Shanghai, China from 1950 to 2016: implications for serogroup B vaccine implementation

Mingliang Chen[1,2,3], Charlene M.C. Rodrigues[2], Odile B. Harrison[2], Chi Zhang[1], Tian Tan[1], Jian Chen[1], Xi Zhang[1], Min Chen[1], Martin C.J. Maiden[2]

[1] Department of Microbiology and Division of Infectious Diseases, Shanghai Municipal Center for Disease Control and Prevention, Shanghai, China;

[2] Department of Zoology, University of Oxford, Oxford, United Kingdom;

[3] Department of Microbiology, Shanghai Institutes of Preventive Medicine, Shanghai, China

Table S1. Analysis of 20 surveys of meningococcal carriage in Shanghai from 1965 to 2016.

Year	Incidence (/100,000)	Number of throat swabs	Isolates (carriage rate, %)	Isolates tested for serogroup	Serogroup (%)			
					A	B	C	Other
1965	139.5	5,367	253 (4.7)	0	ND*	ND	ND	ND
1966	210.7	249	39 (15.6)	13	10 (76.9)	1 (7.7)	1 (7.7)	1 (7.7)
1967	433.8	5,590	1,347 (24.1)	259	188 (72.6)	44 (17.0)	21 (8.1)	6 (2.3)
1970	5.9	818	86 (10.5)	12	6 (50)	0	6 (50)	0
1971	4.2	897	77 (8.6)	9	5 (55.6)	0	4 (44.4)	0
1972	2.7	4,639	111 (2.4)	71	10 (14.1)	43 (60.6)	18 (25.3)	0
1973	2.0	2,025	69 (3.4)	22	3 (13.6)	19 (86.4)	0	0
1974	1.9	1,299	68 (5.2)	0	ND	ND	ND	ND
1975	2.6	855	43 (5.0)	10	0	10 (100)	0	0

1976	3.0	779	52 (6.7)	35	0	29 (82.9)	6 (17.1)	0
1977	6.1	3,561	289 (8.1)	223	3 (1.3)	196 (87.9)	18 (8.1)	6 (2.7)
1978	3.1	2,326	221 (9.5)	184	1 (0.5)	146 (79.3)	11 (6.0)	26 (14.1)
1979	3.2	2,361	177 (7.5)	177	2 (1.1)	123 (69.5)	12 (6.8)	40 (22.6)
2007	0.13	553	11 (2.0)	11	0	11 (100)	0	0
2010	0.01	644	4 (0.6)	4	0	2 (50)	0	2 (50)
2011	0.02	210	1 (0.5)	1	0	1 (100)	0	0
2013	0.02	1,500	17 (1.1)	17	0	16 (94.1)	0	1 (5.9)
2014	0.02	3,330	54 (1.6)	54	0	45 (83.3)	2 (3.7)	5 (9.3)
2015	0.008	360	5 (1.4)	5	0	3 (60)	0	2 (40)
2016	0.01	240	2 (0.8)	2	0	0	0	2 (100)

* ND, not determined.

Table S2. Variants used to assess the strain coverage of serogroup B vaccines

Vaccines	Exact matches	cross-reactive matches	References
Bexsero	fHbp variant 1, NHBA variant 2, NadA variant 8, or PorA-VR2: 4	fHbp variants: 1, 4, 13, 14, 15, 37, or 232; or NadA family: 1 or 2/3	1
Trumenba	fHbp variants: 45 or 55	fHbp variants:1, 4, 13, 14, 15, 16, 19, 21, 23, 24, 25, 30, 47, 76, 87, 180, 187, 252, 276, or 510	2-5
NonaMen	PorA variants: P1.7,16, P1.5-1,2-2, P1.19,15-1, P1.5-2,10, P1.12-1,13, P1.7-2,4, P1.22,14, P1.7-1,1, or P1.18-1,3,6	NA*	6

* NA, not applicable.

Figure S1. Five sequence clusters identified in this study. Sequence types that have no more than 3 different loci with each other, but not assigned in MLST database were assigned to the same cluster. ST-5620 cluster included ST-5542, ST-5620, ST-10395, ST-11040, ST-11041, ST-11044 and ST-12877; ST-5662 cluster was composed of ST-3129, ST-5658, ST-5751, ST-8208, ST-8686, ST-9472, ST-9582, ST-9583, ST-9758, ST-9760, ST-10378, ST-10382, ST-10401, ST-10412, ST-10414, ST-10453, ST-11046, ST-12865, ST-12866, ST-13498, ST-13499, and ST-13504; ST-5819 cluster included ST-5819, ST-8788, ST-10108, ST-11042, ST-11043, ST-11047, ST-12861, ST-12864 and ST-12869; ST-5863 cluster was composed of ST-4830, ST-5636, ST-5863, ST-9587, ST-9588, ST-12860, ST-12862, ST-12867 and ST-12879; ST-9514 cluster included ST-4822, ST-9506, ST-9514, ST-9757 and ST-10398.

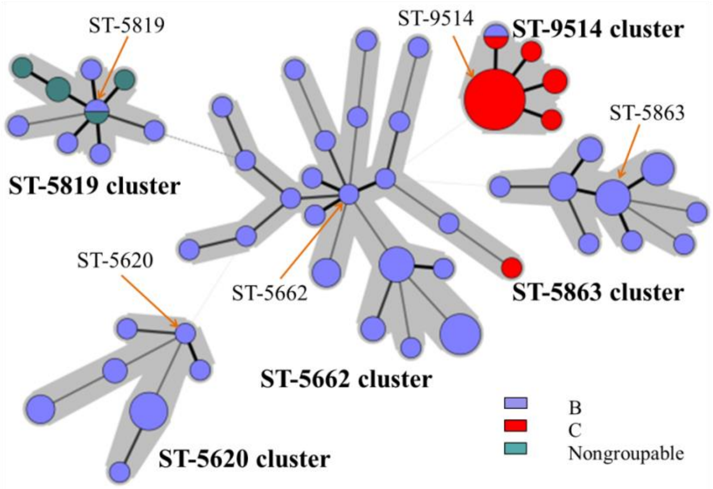

Figure S1

References

1. Charlene, M. C. R. *et al.* Genomic surveillance of 4CMenB vaccine antigenic variants among disease-causing *Neisseria meningitidis* isolates, United Kingdom, 2010–2016. *Emerg Infect Dis* **24**, doi:10.3201/eid2404.171480 (2018).
2. Taha, M. K. *et al.* Bactericidal activity of sera from adolescents vaccinated with bivalent rLP2086 against meningococcal serogroup B outbreak strains from France. *Vaccine* **35**, 1530-1537, doi:10.1016/j.vaccine.2017.01.066 (2017).
3. Ostergaard, L. *et al.* A bivalent meningococcal B vaccine in adolescents and young adults. *N Engl J Med* **377**, 2349-2362, doi:10.1056/NEJMoa1614474 (2017).
4. Lujan, E., Partridge, E., Giuntini, S., Ram, S. & Granoff, D. M. Breadth and duration of meningococcal serum bactericidal activity in healthcare workers and microbiologists immunized with the MenB-FHbp vaccine. *Clin Vaccine Immunol* **24**, e00121-00117, doi:10.1128/CVI.00121-17 (2017).
5. Harris, S. L. *et al.* *Neisseria meningitidis* serogroup B vaccine, bivalent rLP2086, induces broad serum bactericidal activity against diverse invasive disease strains including outbreak strains. *Pediatr Infect Dis J* **36**, 216-223, doi:10.1097/INF.0000000000001399 (2017).
6. Kaaijk, P. *et al.* Preclinical safety and immunogenicity evaluation of a nonavalent PorA native outer membrane vesicle vaccine against serogroup B meningococcal disease. *Vaccine* **31**, 1065-1071, doi:10.1016/j.vaccine.2012.12.031 (2013)
